# Optimizing Hepatocellular Carcinoma Screening: A Comparative Simulated Abbreviated MRI Protocol Study

**DOI:** 10.7759/cureus.78711

**Published:** 2025-02-07

**Authors:** Yasam Preethi, Amber S Papalkar, Sandeep Ponnaganti, Divya Pabbisetti, Ravi Theja Uppuluri, Praharaju Bala Subrahmanyam

**Affiliations:** 1 Department of Radiology, Krishna Institute of Medical Sciences, Secunderabad, IND

**Keywords:** abbreviated protocol, cirrhosis, dynamic contrast-enhanced mri (dce-mri), hbv, hepatocellular carcinoma, hepatocellular carcinoma screening, li-rads, mri liver, onco-imaging

## Abstract

Background

Patients with risk factors such as viral hepatitis-induced liver cirrhosis, advanced-stage primary biliary cirrhosis, hereditary hemochromatosis, metabolic-associated fatty liver disease, and alcoholic liver disease are more likely to develop hepatocellular carcinoma (HCC). Most HCC patients have advanced-stage disease unresponsive to treatment. Therefore, avoiding or treating viral infections and early detection through routine surveillance, such as repeated liver ultrasonography, are the most effective ways to reduce HCC-related mortality. However, as the sensitivity of ultrasound is low, many centers use contrast-enhanced CT and/or MRI for HCC surveillance. This study aimed to evaluate the per-lesion performance of simulated abbreviated non-contrast MRI (aNC-MR) compared to conventional complete MRI (cMR) for screening high-risk patients for HCC. In addition, it aimed to assess the per-lesion performance of simulated abbreviated dynamic multiphase contrast-enhanced MRI (aDCE-MRI) protocol compared to cMR for screening high-risk patients for HCC.

Methodology

This retrospective study conducted at a single tertiary care center included patients with liver disease at high risk for HCC over five years. These patients underwent cMR for screening. The aNC-MR protocol included diffusion-weighted imaging and T2-weighted imaging, while the aDCE-MR protocol included only the non-contrast T1 fat-saturated and dynamic post-contrast sequence. Two independent radiologists, blinded to the original cMR diagnosis, assessed two abbreviated protocol image sets, namely, an aDCE-MR set and an aNC-MR set. Each lesion was categorized as suspicious for HCC or suitable for interval screening on the aNC-MR protocol set. On the aDCE-MR image set, the lesions were designated using Liver Imaging Reporting and Data System categorization.

Results

Of a total of 125 lesions, 110 were evaluated and characterized using the simulated aNC-MR protocol, as 15 lesions could not be seen on aNC-MR, giving the aNC-MR protocol an overall sensitivity of 88%. On a per-lesion basis, the aNC-MR protocol compared with cMR demonstrated a negative predictive value of 96.4% and a specificity of 96.4%. Overall, 125 lesions were evaluated and characterized using the simulated aDCE-MR protocol. On a per-lesion basis, the aDCE-MR protocol compared with cMR demonstrated an negative predictive value of 100% and a specificity of 98.6%. Comparable results were observed in patients with hepatic steatosis.

Conclusions

The aNC-MR protocol can significantly reduce the cost and time required for MRI screening in patients at high risk for HCC. This protocol was equally effective in patients with hepatic steatosis and suboptimal ultrasound results, making it a viable and efficient screening modality.

## Introduction

Patients with a recognized risk factor are more likely to develop the majority of hepatocellular carcinomas (HCCs). Globally, the most prevalent risk factor for HCC is viral hepatitis-induced liver cirrhosis. Advanced-stage primary biliary cirrhosis, hereditary hemochromatosis, metabolic-associated fatty liver disease, and alcoholic liver disease are additional risk factors [1,2]. The majority of patients with HCC are found to have advanced-stage carcinoma unresponsive to possible treatments for the disease [3]. Therefore, avoiding or treating viral infections and detecting HCC early through routine surveillance are the most efficient methods to lower the death rate of HCC.

The European Association for the Study of the Liver, the American Association for the Study of Liver Diseases, and the Asian Pacific Association recommend biannual hepatic ultrasonography for patients with risk factors for HCC surveillance [4-7]. In the majority of cases, ultrasound is the method of choice for HCC surveillance as it is readily available and reasonably priced. Nonetheless, two meta-analyses of 26 distinct trials revealed that the pooled per-patient sensitivity for HCC diagnosis in high-risk individuals was 60-63% [8,9]. Many centers use CT and/or MRI for HCC surveillance, though not as the first line. Ultrasound of the liver with alpha-fetoprotein (AFP) remains the main modality for screening, even though ultrasound has a limited sensitivity. The greatest reported sensitivity for diagnosing HCC is 81% with contrast-enhanced MRI [9].

The American College of Radiology introduced the Liver Imaging Reporting and Data System (LIRADS) in 2011 which was subsequently updated. It is designated to standardize the radiologic diagnosis of HCC, thereby allowing for consistent terminology, decreasing variability in reporting, as well as better communication with referring physicians [10,11]. Five major criteria are evaluated in assigning the LI-RADS category to a liver lesion/observation, namely, size, arterial phase hyperenhancement, washout appearance, enhancing capsule, and threshold growth. Multiple ancillary features, including T2 hyperintensity and diffusion restriction, can be used to upgrade the LI-RADS category [10,11].

Thus, we conducted this study to evaluate the per-lesion performance of two sets of simulated abbreviated MRI (AMRI) protocols, that is, the simulated abbreviated non-contrast MRI (aNC-MR) and simulated abbreviated dynamic multiphase contrast-enhanced MRI (aDCE-MRI) protocols, compared to conventional complete MRI (cMR) for screening high-risk patients for HCC.

## Materials and methods

This retrospective study was conducted at a single tertiary care center and was approved by the Institute Ethics Committee.

Inclusion criteria

The study included patients with cirrhosis or chronic hepatitis B/C who underwent contrast-enhanced upper abdominal MRI between July 1, 2018, and June 30, 2023, and had at least one focal liver lesion. The diagnosis of cirrhosis was confirmed by cross-sectional imaging or biopsy. The imaging-based diagnosis of HCC is globally adopted, ensuring the study’s results reflect real-world scenarios. A total of 62 patients with 125 liver lesions were included in the study.

Exclusion criteria

Patients with a history of primary extrahepatic malignancy, metastatic disease, or treated HCC were excluded from our study. Patients with prior LI-RADS 4 or 5 lesions were excluded to simulate a true screening environment.

Study protocol

Diffusion-weighted imaging (DWI) and T2 and T1-weighted imaging were evaluated as part of the simulated aNC-MR protocol. Dynamic multiphase contrast-enhanced MRI sequences (using gadodiamide contrast, 0.1 mmol/kg) were assessed as part of the simulated aDCE-MR protocol. Conventional complete MRI included both sets (aNC-MR + aDCE-MR). The standard MRI liver protocol at our center is mentioned in Table 1.

**Table 1:** MRI protocol for the liver. TR: repetition time; TE: time to echo; T2WI: T2-weighted imaging; T1WI: T1-weighted imaging; DWI: diffusion-weighted imaging; CET1WI: contrast-enhanced T1-weighted imaging

Parameters on 3 Tesla	T2WI	T1WI	DWI	DIXON	CET1WI
TR (ms)	1,000	10	1,000	500	500
TE (ms)	80	2.3	55.33	50	50
Flip angle (degrees)	90	15	90	10	10
Slice thickness (mm)	5	5	5	1.8	1.6
Reconstruction interval (mm)	1	1	1		
Acquisition matrix	240 × 151	208 × 119	124 × 100	172 × 114	216 × 170
Signal averages	1	1	2	1	1
b-values (s/mm^2^)	-	-	300/500/1,000	-	-
Acquisition time (s)	110	110	270	12	100

Study methodology

Two independent experienced abdominal radiologists assessed the three sets of images, namely, aNC-MR, aDCE-MR, and cMR. Randomly distributed MR image sets were given to both radiologists blinded to the presence or location of any liver lesions and the final imaging results. The observers analyzed the three sets of images in three different sessions, separated by at least six weeks to reduce recall bias. On non-contrast image sets, MRI findings favoring HCC included (1) high signal intensity lesions on T2 weighted imaging, except for very high signals indicating hemangiomas or cysts; and (2) diffusion restriction on DWI, i.e., high signal intensity on DWI with reversal on the apparent diffusion coefficient map. If any of these findings were noted in focal hepatic lesions, the lesions were deemed “suspicious” and required contrast-enhanced MRI. A maximum of five lesions per patient were considered. On aDCE-MRI sets, focal hepatic lesions were assigned an LI-RADS score based on specific imaging features, including arterial phase non-rim hyperenhancement, non-peripheral washout, and enhancing capsule appearance.

After an independent review, the cMR findings of each study were re-evaluated in consensus by the two reviewers regarding the presence of findings favoring HCC. Thus, this retrospective study evaluated the diagnostic performance of simulated AMRI to detect HCC in a cross-sectional study design.

Statistical analysis

Data entry was done using MS Excel and was statistically analyzed using SPSS version 25 (IBM Corp., Armonk, NY, USA). Descriptive statistical analysis was performed to explore the distribution of several categorical and quantitative variables. Sensitivity, specificity, and predictive values were calculated for the aNC-MR and aDCE-MR protocols and compared with the cMR protocol. Kappa statistics were used to assess agreement between the two procedures. A p-value less than 0.05 was considered statistically significant.

## Results

Demographics

Our study population comprised 62 patients (males, 53; females, 9) with a mean age of 56.77 years (minimum, 38 years; maximum, 75 years). Most patients with HCC lesions were in the 65-75-year age group. The most prevalent risk factor in HCC patients was found to be alcoholic liver disease with 56.3% (18/28), followed by equal proportions of hepatitis B and C.

Overall lesion characterization

Of the 62 patients at high risk for HCC, 54.8% (n = 34) had benign lesions and 45.2% (n = 28) had lesions that turned out to be HCC on cMR. A total of 125 lesions were seen and characterized on cMR. Of the 125 lesions detected on the cMR protocol, 15 could not be seen on the simulated aNC-MR protocol, giving the aNC-MR protocol an overall sensitivity of 88%. The remaining 110 lesions seen on the aNC-MR protocol were detected and characterized on the cMR protocol. Per-lesion analyses were performed to calculate the sensitivities, specificities, positive predictive value, and negative predictive values for simulated abbreviated protocols compared to conventional complete MRI protocol. For analytical purposes, the lesions were categorized as lesions measuring >20 mm, 10-20 mm, and <10 mm in maximal dimensions. The overall lesion characterization on aNC-MR and aDCE-MR protocols is presented in Table 2.

**Table 2:** Overall lesion characterization on the aNC-MR and aDCE-MR protocols. aNC-MR: simulated abbreviated non-contrast MRI; aDCE-MR: simulated abbreviated dynamic contrast-enhanced MRI; cMR: complete MRI

Comparison of aNC-MR with cMR for 110 visualized lesions						
		cMR			Total	
		Malignant	Benign			
aNC-MR	Suspicious	52	2		54	
	Benign	2	54		56	
Total		54	56		110	
Comparison of aDCE-MR with cMR for 125 lesions						
		cMR			Total	
		Malignant	Benign			
aDCE-MR	Malignant	53	0		53	
	Benign	1	71		72	
	Total	54	71		125	
Comparison of aNC-MR with cMR for lesions measuring <10 mm						
Lesion size			cMR			Total
			Malignant	Benign		
<10 mm	aNC-MR	Suspicious	3	1		4
		Benign	2	18		20
	Total		5	19		24
Comparison of aDCE-MR with cMR for lesions measuring <10 mm						
Lesion size			cMR			Total
			Malignant	Benign		
<10 mm	aDCE-MR	Malignant	4	0		4
		Benign	1	28		29
	Total		5	28		33
Comparison of aNC-MR with cMR for lesions measuring 10–20 mm						
Lesion size			cMR			Total
			Malignant	Benign		
10–20 mm	aNC-MR	Suspicious	11	1		12
		Benign	0	23		23
	Total		11	24		35
Comparison of aDCE-MR with cMR for lesions measuring 10–20 mm						
Lesion size			cMR			
			Malignant	Benign		Total
10–20 mm	aDCE-MR	Malignant	11 0	0		11
		Benign	0	30		30
	Total		11	30		41
Comparison of aNC-MR with cMR for lesions measuring >20 mm						
Lesion size			cMR			Total
			Malignant	Benign		
>20 mm	aNC-MR	Suspicious	38	0		38
		Benign	0	13		13
	Total		38	13		51
Comparison of aDCE-MR with cMR for lesions measuring >20 mm						
Lesion size			cMR			Total
			Malignant	Benign		
>20 mm	aDCE-MR	Malignant	38	0		38
		Benign	0	13		13
	Total		38	13		51

Overall Lesion Analysis on the aNC-MR Protocol

A total of 110 lesions were seen and characterized on the simulated aNC-MR protocol and compared with cMR. On a per-lesion basis, the aNC-MR protocol had a specificity of 96.4%, positive predictive value of 96.3%, negative predictive value of 96.4%, and sensitivity of 96.3%, with a p-value of less than 0.01 (highly significant) (Table 3).

**Table 3:** Statistical analysis of the aNC-MR protocol compared to cMR for 110 visualized lesions. aNC-MR: simulated abbreviated non-contrast MRI; cMR: complete MRI

Statistics	Value	95% CI	P-value
Sensitivity	96.30%	87.25% to 99.55%	0.000
Specificity	96.43%	87.69% to 99.56%	0.000
Positive predictive value	96.30%	86.95% to 99.02%	0.000
Negative predictive value	96.43%	87.38% to 99.06%	0.000
Accuracy	96.36%	90.95% to 99.00%	0.000

Overall Lesion Analysis on the aDCE-MR Protocol

Overall, 125 lesions were evaluated and characterized with the simulated aDCE-MR protocol and compared with cMR. Per-lesion, aDCE-MRI had a sensitivity of 98.1%, specificity of 100%, positive predictive value of 100%, and a negative predictive value of 98.6%, with a p-value of less than 0.01 (highly significant) (Table 4).

**Table 4:** Statistical analysis of the aDCE-MR protocol compared to cMR for 125 lesions. aDCE-MR: simulated abbreviated dynamic contrast-enhanced MRI; cMR: complete MRI

Statistics	Value	95% CI	P-value
Sensitivity	98.15%	90.11% to 99.95%	0.000
Specificity	100.00%	94.94% to 100.00%	0.000
Positive predictive value	100.00%	93.28% to 100.00%	0.000
Negative predictive value	98.61%	91.06% to 99.80%	0.000
Accuracy	99.20%	95.62% to 99.98%	0.000

Thus, sensitivities and negative predictive values of the aNC-MR and aDCE-MR protocols were on par with cMR in terms of accuracy with a statistically significant p-value.

Lesions measuring >20 mm

aNC-MR Protocol

Of 51 lesions measuring >20 mm, 38 were characterized as suspicious on the aNC-MR set, all of which turned out to be LI-RADS 4, 5, and M lesions on cMR with 100% sensitivity, 100% specificity, 100% positive predictive value, and 100% negative predictive value (Table 5).

**Table 5:** Statistical analysis of the aNC-MR protocol compared to cMR for lesions measuring >20 mm. aNC-MR: simulated abbreviated non-contrast MRI; cMR: complete MRI

Statistics	Value	95% CI	P-value
Sensitivity	100.00%	90.75% to 100.00%	0.000
Specificity	100.00%	75.29% to 100.00%	0.000
Positive predictive value	100.00%	90.75% to 100.00%	0.000
Negative predictive value	100.00%	75.29% to 100.00%	0.000
Accuracy	100.00%	93.02% to 100.00%	0.000

aDCE-MR Protocol

Of 51 lesions measuring >20 mm, 38 were characterized as LI-RADS 4, 5, and M on the aDCE-MR set, all of which turned out to be LI-RADS 4 or 5, and M lesions on cMR with 100% sensitivity, 100% specificity, 100% positive predictive value, and 100% negative predictive value (Table 6).

**Table 6:** Statistical analysis of the aDCE-MR protocol compared to cMR for lesions measuring >20 mm. aDCE-MR: simulated abbreviated dynamic contrast-enhanced MRI; cMR: complete MRI

Statistics	Value	95% CI	P-value
Sensitivity	100.00%	90.75% to 100.00%	0.000
Specificity	100.00%	75.29% to 100.00%	0.000
Positive predictive value	100.00%	90.75% to 100.00%	0.000
Negative predictive value	100.00%	75.29% to 100.00%	0.000
Accuracy	100.00%	93.02% to 100.00%	0.000

For lesions measuring >20 mm, sensitivity and negative predictive value were 100% with a p-value of less than 0.01 (highly significant) on both simulated aNC-MR and aDCE-MRI protocols.

Lesions measuring 10-20 mm

aNC-MR Protocol

Of 35 lesions measuring 10-20 mm, 11 were categorized as suspicious on the aNC-MR set, all of which turned out to be LI-RADS 4 or 5 and M lesions on cMR, while one lesion characterized as benign on aNC-MRI was classified as LR-4 on cMR due to its enhancement characteristics, thereby giving 100% sensitivity, 95.8% specificity, 100% negative predictive value, and 91.6% positive predictive value (Table 7).

**Table 7:** Statistical analysis of the aNC-MR protocol compared to cMR for lesions measuring 10-20 mm. aNC-MR: simulated abbreviated non-contrast MRI; cMR: complete MRI

Statistics	Value	95% CI	P-value
Sensitivity	100.00%	71.51% to 100.00%	0.000
Specificity	95.83%	78.88% to 99.89%	0.000
Positive predictive value	91.67%	61.76% to 98.68%	0.000
Negative predictive value	100.00%	85.18% to 100.00%	0.000
Accuracy	97.14%	85.08% to 99.93%	0.000

aDCE-MR Protocol

Of 41 lesions measuring 10-20 mm, 11 belonged to LI-RADS 4 or 5 and M on the aDCE-MR set, all of which turned out to be LI-RADS 4 or 5 and M lesions on cMR with 100% sensitivity, specificity, positive predictive value, and negative predictive value (Table 8).

**Table 8:** Statistical analysis of the aDCE-MR protocol compared to cMR for lesions measuring 10-20 mm. aDCE-MR: simulated abbreviated dynamic contrast-enhanced MRI; cMR: complete MRI

Statistics	Value	95% CI	P-value
Sensitivity	100.00%	71.51% to 100.00%	0.000
Specificity	100.00%	88.43% to 100.00%	0.000
Positive predictive value	100.00%	71.51% to 100.00%	0.000
Negative predictive value	100.00%	88.43% to 100.00%	0.000
Accuracy	100.00%	91.40% to 100.00%	0.000

For lesions measuring 10-20 mm, sensitivity and negative predictive value were 100% with a p-value of less than 0.01 (highly significant) on both simulated aNC-MR and aDCE-MRI protocols.

Lesions measuring <10 mm

aNC-MR Protocol

Of 24 lesions measuring <10 mm, three were categorized as suspicious on the aNC-MR set, all of which turned out to be LI-RADS 4 or 5 and M lesions on cMR. One lesion characterized as benign on aNC-MRI was classified as LR-4 on cMR due to enhancement characteristics (false negative), thereby giving a sensitivity of 60%, specificity of 94.7%, positive predictive value of 75%, and negative predictive value of 90% with a p-value of 0.003 (significant) (Table 9).

**Table 9:** Statistical analysis of the aNC-MR protocol compared to cMR for lesions measuring <10 mm. aNC-MR: simulated abbreviated non-contrast MRI; cMR: complete MRI

Statistics	Value	95% CI	P-value
Sensitivity	60.00%	14.66% to 94.73%	0.003
Specificity	94.74%	73.97% to 99.87%	0.003
Positive predictive value	75.00%	28.11% to 95.84%	0.003
Negative predictive value	90.00%	75.37% to 96.36%	0.003
Accuracy	87.50%	67.64% to 97.34%	0.003

aDCE-MR Protocol

Of 33 lesions measuring <10 mm, four lesions were categorized as LI-RADS 4 or 5 and M on aDCE-MRI, all of which turned out to be LI-RADS 4 or 5 and M lesions on cMR, while one lesion characterized as LR-3 on aDCE-MRI was classified as LR-4 on cMR due to ancillary features (false negative), giving a sensitivity of 80%, specificity of 100%, positive predictive value of 100%, and negative predictive value of 96.5% with a p-value of less than 0.01 (highly significant) (Table 10).

**Table 10:** Statistical analysis of the aDCE-MR protocol compared to cMR for lesions measuring <10 mm. aDCE-MR: simulated abbreviated dynamic contrast-enhanced MRI; cMR: complete MRI

Statistics	Value	95% CI	P-value
Sensitivity	80.00%	28.36% to 99.49%	0.000
Specificity	100.00%	87.66% to 100.00%	0.000
Positive predictive value	100.00%	39.76% to 100.00%	0.000
Negative predictive value	96.55%	82.91% to 99.39%	0.000
Accuracy	96.97%	84.24% to 99.92%	0.000

**Figure 1:**
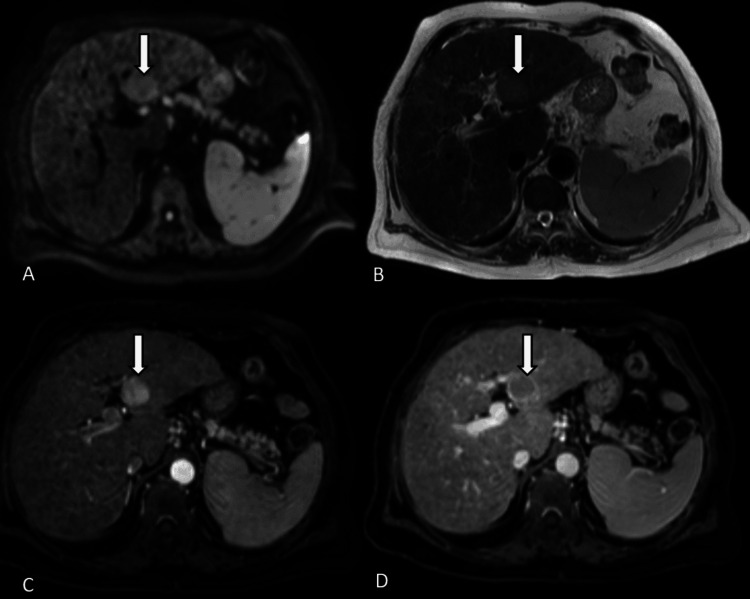
Axial MRI for a patient on HCC surveillance depicts a lesion in the left lobe of the liver (arrow). The lesion is hyperintense on DWI (A) and hyperintense on T2WI (B). It was characterized as suspicious on aNC-MRI. Further, the lesion shows typical arterial-phase hyperenhancement (C) with washout and peripheral capsule on later phases of the multiphase post-contrast study (D), thus making it a LI-RADS LR-5 lesion. DWI: diffusion-weighted imaging; T2WI: T2-weighted imaging; aNC-MR: simulated abbreviated non-contrast MRI; LI-RADS: Liver Imaging Reporting and Data System

**Figure 2:**
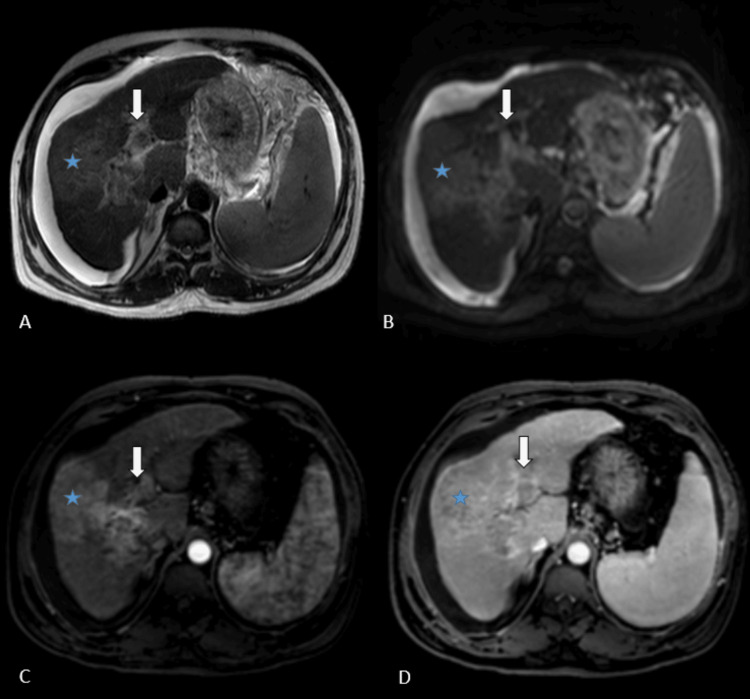
Composite axial MRI of a patient on HCC surveillance depicts a lesion in the right lobe (*) with tumor thrombus in the portal vein (arrow). The lesion is hyperintense on T2WI (A) and hyperintense on DWI (B). It was characterized as suspicious on the aNC-MR protocol requiring cMR for further evaluation. Confluent enhancing thrombus is seen in the portal vein (C), showing washout on later phases of the multiphase post-contrast study (D) conforming the diagnosis of HCC with portal vein tumor thrombus. DWI: diffusion-weighted imaging; T2WI: T2-weighted imaging; aNC-MR: simulated abbreviated non-contrast MRI; cMR: complete MRI; HCC: hepatocellular carcinoma

**Figure 3:**
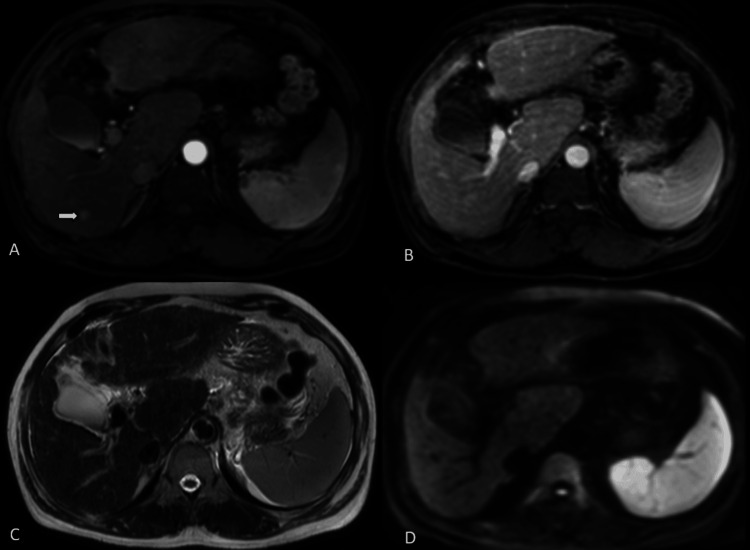
Composite axial MRI of a patient with chronic liver disease shows an arterial-phase hyperenhancing subcentimeter-sized lesion (arrow, A). This lesion was isointense to the liver parenchyma on delayed-phase (B), T2-weighted (C), and diffusion-weighted (D) images, and thus was not seen on the aNC-MR protocol. This lesion was categorized as LR-3 on cMR and required further imaging in less than six months. aNC-MR: simulated abbreviated non-contrast MRI; cMR: complete MRI

The composite MRI scans below illustrate the appearance of typical HCC (Figure 1) and HCC with tumor invasion into the portal vein (Figure 2) across different MR sequences included in the aNC-MR and aDCE-MR protocols. Additionally, an arterial-phase subcentimeter hyperenhancing lesion, which was not visible on the aNC-MR protocol, is shown in Figure 3.

## Discussion

Internationally accepted standards recommend biannual ultrasound screening for HCC, which is crucial for early diagnosis in high-risk patients. However, suboptimal or non-diagnostic ultrasound scans, such as those in patients with high body mass index, fatty liver, cirrhosis, and multiple benign liver lesions, limit the use of ultrasound surveillance. There are currently no guidelines that address how to screen these patients when ultrasonography is subpar [7,12].

Chan et al. [13] retrospectively assessed 188 patients at high risk for HCC with an aNC-MRI protocol. They demonstrated a high specificity of 93%, a negative predictive value of 97%, and a sensitivity of 85%. Our study noted comparable results for lesions measuring more than 20 mm and is in strong agreement with earlier findings. Khatri et al. [14] retrospectively assessed 93 patients at high risk for HCC using an aDCE-MRI protocol and demonstrated a sensitivity of 88%. This strongly agrees with our aDCE-MRI study, which had a sensitivity of 98.1%. Han et al. [15] retrospectively assessed 247 patients at high risk for HCC, of whom 175 had HCC and 72 were control with an aNC-MRI protocol and demonstrated per-lesion specificity of 82% and sensitivity of 84%. Our study is in concordance with these findings.

Our study also strongly agreed in terms of sensitivity and negative predictive value with the findings of Lee et al. [16], who retrospectively assessed 164 patients at high risk for HCC with an aNC-MRI protocol, and Tillmann et al. [17] retrospectively evaluated 79 patients, of whom 13 were found to have HCC. Besa et al. [18] retrospectively assessed 174 patients, of whom 62 were found to have HCC, and 112 as controls at high risk for HCC with an aDCE-MRI protocol. They demonstrated a negative predictive value of 98.6% with a sensitivity of 98.1%. Our study showed comparable results and is in agreement with previous findings.

Overall sensitivities declined for lesions smaller than 20 mm. However, they were still greater than the pooled sensitivity by ultrasonography, which had a 59.3% sensitivity according to a meta-analysis by Hanna et al. [19]. Lesions smaller than 10 mm (60%) had a lower sensitivity on aNC-MR than lesions larger than 10 mm (100%). Based on the Milan criteria, this scored favorably with ultrasound’s aggregate sensitivity of 47% for identifying early-stage HCC [14,15].

Although contrast-enhanced MRI is the imaging method of choice for HCC diagnosis and staging, current practice guidelines do not advocate the same for HCC surveillance due to long exam duration, limited access, and costs [9,20,21]. aNC-MR and aDCE-MR protocols are being evaluated as an alternative to ultrasonography for HCC surveillance as they rely on a few selected MRI sequences. The main objective of abbreviated protocols is to achieve acceptable diagnostic performance for HCC detection while decreasing acquisition time and cost [21].

There is significant variation in how HCC appears on DWI sequences, which is still essential for hepatic non-contrast MR imaging [22,23]. Although DWI is not very specific or sensitive for HCC [24,25], it is sensitive for malignancy and plays a significant role when hepatic steatosis is present. The evaluation of liver lesions with and without hepatic steatosis did not differ in terms of sensitivity or negative predictive value. Following an examination in conjunction with the T1-weighted and T2-weighted sequences, the screening scan was taken into consideration for additional contrast-enhanced imaging in our research if a lesion showed restricted diffusion.

The per-lesion sensitivity and negative predictive value of aNC and aDCE-MR protocols for lesions larger than 10 mm was 100%, indicating that these shortened MRI procedures might be a suitable substitute for HCC surveillance in at-risk individuals. However, aDCE-MR cannot be used in patients who have concomitant renal impairment and is associated with an additional cost for contrast. On the other hand, for the majority of patients undergoing HCC surveillance, the aNC-MR strategy is practical, economical, and time-efficient [26].

In our study, 15 lesions, all of which measured less than 10 mm, could not be visualized on aNC-MR, resulting in reduced sensitivities. These lesions were seen as arterial-phase hyperenhancing foci on aDCE-MR and were characterized as LR-3 on cMR, requiring a repeat scan in three to six months [10].

In this study, we assessed the per-lesion detection accuracy for HCC via independent readings by two radiologists, simulating the aNC-MR protocol in a screening context. Additionally, as our readers were blinded to prior studies, the results are only partially representative of clinical practice and represent a challenging scenario. Given the retrospective nature of our study, there can be a selection bias in the patient sample. In the actual surveillance environment, the prevalence of HCC will be lower than it was in our study. Moreover, our study was conducted at a single tertiary care center with a limited sample size, impacting the generalizability of our findings. Lack of AFP correlation in our study could also be a potential limitation. To improve sensitivity and specificity and thereby enhance reader confidence, an initial baseline contrast-enhanced liver MRI may be suggested, followed by a biannual serial aNC-MRI for comparison. Future studies for sensitivity and specificity estimates of AMRI protocols are recommended in a screening population and should be independent of disease prevalence with a multicenter approach. Future research should focus on creating risk-stratified AMRI protocols possibly incorporating AFP values, evaluating cost-effectiveness, and comparing AMRI protocols with ultrasound to determine the best surveillance strategies.

## Conclusions

AMRI protocols, especially non-contrast MRI are relatively inexpensive, safe, easy to perform, and less time-consuming compared to cMRI, making them a potential tool for HCC surveillance. The aNC-MR protocol can significantly lower the cost and time required for screening high-risk patients, proving equally effective in patients having hepatic steatosis with suboptimal ultrasound as well as in patients who are unable to tolerate contrast agents, such as in patients with severe allergy or concomitant renal failure. Furthermore, these protocols can impact clinical workflow by reducing patient waiting times and optimizing imaging resources in routine practice.
